# The Heart after the War

**Published:** 1918-08

**Authors:** 


// BUFFALO MEDICAL JOURNAL

A Monthly Review of Medicine and Surgery

EDITOR AND PUBLISHER, DR. A. L. BENEDICT, 228 Summer St., corner of Elmwood Ave., Buffalo, (Address for all communications. Please make personal and telephone calls before 1 P. M.)

Third, in age, on the western continent. Independent, but supports professional organizations. News limited mainly to Western N. Y. and Penn.

Subscription $2.00 a year; $3.00 for two years in advance. Changes and errors in address or failure or duplication of delivery, should be reported immediately.

Yearly Volume 74 AUGUST, 1918 Number 1

### The Heart After the War.

Reports are unanimous as to the general health and increase in physical development and resistance of men suddenly transferred from civil to military life. Even if the former habits have not included much exercise, the great majority of men even of middle age, as in the Medical Reserve Corps, have readily adapted themselves to the average increase in physical demands. It should be considered, however, that the pendulum has swung back from a period with industrial and social tendencies to sedentary occupations with little exercise to a very general interest in extra-vocational physical training of one kind or another. Thus, the outbreak of the present war, probably found our country with at least as great an average muscular development as at the time of the Civil War when a relatively larger proportion of the population was engaged in agriculture and other occupations necessitating physical strength, and with a much higher average of physical health and general resistance, which are by no means synonymous with muscular strength.

Barring actual discoverable lesions, as of the heart, vessels, lungs, kidneys, thyroid, liver, etc., and deformities, mutilations, defects of special senses, etc., which more or less definitely exclude from military service though not necessarily involving the general health, the vast majority of failures to stand the strain of military life may be placed under the old term of irritable heart or under the newer English term of Effort Syndrome, meaning that the heart and vascular system and, to a certain extent, also, the respiratory functions, do not easily meet the sudden increased strain so that, without demonstrable disease and without necessarily preventing the performance of ordinary civil duties of the individual, even if he has broken down under military training or has passed the period of training and has broken down in actual service at the front, he is useless as a soldier. While no statistics have been published either for the British or our own troops, it may be said that the Effort Syndrome involves an appreciable though small proportion of soldiers and that the last sentence requires qualification as about 60% of those involved are returned to duty after rest and a course in graduated exercises of the general nature of calisthenics and setting up exercises, drugs and rest treatment in the limited sense having been found to be useless.

Medical men scarcely need to be told that the heart normally hypertrophies very readily to meet increased demands but it has been a surprise to many, that this response occurs, either in athletics or military training very rapidly, even in a month, in the vast majority of all cases, even when the previous habits have been strictly sedentary and when only the most rudimentary precautions have been taken to prevent undue and sudden strain; also that even the failures have seldom resulted in either temporary incapacity or permanent damage of any degree.

Pierre Nadal, in a series of articles in the Jour. de Med. de Bordeaux, has considered especially the physical training of "intellectuals." While, in the main, he says nothing that is not obvious as to general principles and that is not included in descriptions of calisthenic and especially setting up exercises, including the special adaptations to the Effort Syndrome, some of his points might be overlooked. The necessity of developing articular suppleness as well as muscular strength; of securing instant obedience to commands, of properly coordinating movements and of excluding certain habits of improper coordination and inhibition, of psychic and psycho-neural as well as gross muscular response, etc., might be missed especially by one attacking the subject without actual experience. One detail of his seems especially important: catching the breath, on attempting a difficult and especially a straining task, is not really a normal reflex but one which should be overcome by training as it obviously—when we stop to reflect—diminishes the supply of oxygen and immediate elimination of waste and imposes additional cardio-vascular strain, at the very time when the most favorable conditions are demanded. Another point which might be neglected and which, we believe, has been in some military commands, is the importance of nutrition and hygiene concomitant with physical development. A large part of the minor morbidity of camps, predisposing also to serious infections, especially with pneumonia and influenza, has been ascribed by military critics to chill following the inevitable perspiration of drills, especially when the routine prevented bathing and change of clothing for the major part of the day or when the soldier was, on account of fatigue and lack of time that could be spared from sleep, tempted to sleep in damp underwear.

We venture to predict that the health and resistance of our troops, even without attention to these details, will be much better for the second winter, even for the first winter in service of men trained during the whole summer and fall, and that the routine of the front, when exercise is mostly combined with the psychic stimulation of actual fighting and when periods of service alternate with those of rest and attention to hygienic details, will result in less morbidity than cantonment life. Thus the general prediction may be made that the transformation of a civilian young male adult population into soldiers, will result in very little loss, after that incident to the weeding out of genuine defectives, though most of these are not defectives according to the demands of the ordinary vocations to which they have been adapted. Even this is not a military loss of serious moment, since it is not at all likely that we shall be required to raise even half of the theoretic ''militia'' of 10% of the total population and since a large percentage of the adult population must, by any country that does not rely to an undue degree upon the labor of women and children or of enslaved aliens, be retained in civil occupations, even if we look no farther ahead than military necessity.

In civil practice—and undoubtedly military—physiologic hypertrophy of the heart is not merely a theory nor even a demonstration from the standpoint of general pathology. It is something readily detected by clinical methods—perhaps most convincingly by the X-ray, but also by percussion, either ordinary or auscultatory. In particular the left border of the heart of an athlete or of one who has done much muscular work, is 1-2 c.m. outside the line established by one who has not used his muscles to any degree. This difference can even be noted in the same individual after a few months, perhaps a few weeks, of exercise. The pathologic principle (pathologic only in the sense that the matter is usually included in the teaching of pathology) that the heart readily hypertrophies in response to stimuli has an unfortunate corollary, that the hypertrophied heart does not so readily atrophy, without actual degenerative changes, when the continued stimulus is withdrawn. Moreover, we must reckon with a general hypertrophy of the vascular apparatus, not so easily demonstrated even histologically except for the larger trunks and almost undemonstrable clinically. Even if it were conceded that the vessels participated only functionally in the increased circulatory response, we should still have to consider that the viscera and other organs have adapted themselves to a greater circulatory activity, including all that this implies from the standpoint of respiration, and that the reflex response of all organs, especially those of nutrition and elimination, have become attuned to grosser excito-reflex impressions, and that the regulation of the physical apparatuses in general has been effected by innervational discharges from which comparatively little has been subtracted for intellectual work.

The actual lesions and functional disturbances to which ex-athletes and those who have changed from muscular to brain work, or from active to sedentary occupations, are liable, have been described in many articles though the profession and hygienists generally do not yet seem to realize fully the importance of this subject. It would be out of place in an editorial article, to enter into details, but it is important that the general subject should receive due attention in advance of the immediate need of considering the personal health of the two or three million men who will be restored to civil life in what we hope will be the comparatively near future. This subject should also receive attention from the standpoint of the discussion as to universal military training, for it is something more than an aggregation of personal needs for guidance. It involves sociologic problems that tend to ramify. Off-hand, we are inclined to believe that the only solution of the problem, whether for the athlete, the muscle worker who retires or who changes his vocation at an early age, or for the soldier adjusting himself to civil life, is a moderate continuance of physical work and an avoidance of the toxaemias of alcohol, tobacco, and of preventable diseases and unhygienic modes of living. The first factor is more important than it seems. It is easy to advise, but difficult to carry out without involving a large amount of wasted efficiency unless the physical work can be rendered of practical value in some way and right here, the problem begins to ramify along all sorts of industrial, sociologic and almost political lines. But it, as well as the other factors, must be made operative, or else great harm will result to many individuals and a not negligible amount to posterity.

